# A Novel Approach for the Determination of the Ge Isotope Ratio Using Liquid–Liquid Extraction and Hydride Generation by Multicollector Inductively Coupled Plasma Mass Spectrometry

**DOI:** 10.1021/acs.analchem.1c02491

**Published:** 2021-09-30

**Authors:** Jakub Karasiński, Andrii Tupys, Ludwik Halicz, Ewa Bulska

**Affiliations:** † Faculty of Chemistry, Biological and Chemical Research Centre, University of Warsaw, Żwirki i Wigury 101, Warsaw 02-093, Poland; ‡ Geological Survey of Israel, 32 Y. Leybowitz St., Jerusalem 9692100, Israel

## Abstract

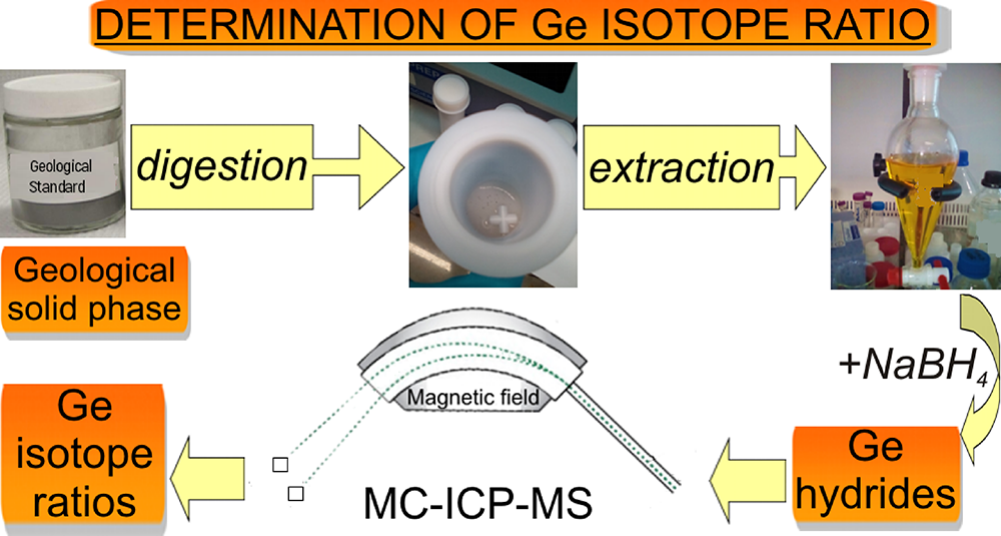

In this work, a method for the accurate and precise determination of the Ge isotope ratio in synthetic water and natural samples of geological origin using multicollector inductively coupled plasma mass spectrometry (MC-ICPMS) with hydride generation was developed. The method was based on the liquid–liquid extraction of Ge to eliminate all elements affecting the generation of germanium hydrides. The standard-sample bracketing method was used to correct instrumental bias. Registration of analytical signal in time-resolved mode gave way to choose signals with best parameters and improved the precision of the results. Controlling the pH by using acetic buffer boosted the sensitivity by nearly five times in comparison to hydride generation methods suggested by other authors. The newly developed method is much simpler and quicker, does not need laborious Ge separation with ion-exchange resins, and thanks to its superior sensitivity, allows measurements of the Ge isotopic ratio in materials with relatively low Ge content. Delta values of the ^74^Ge/^70^Ge isotope ratio were measured in standard reference materials for which reference values were available in the GeoREM database. We demonstrated that the accuracy and precession of this method are equally good or better than methods proposed by other authors.

Germanium is a trace element in the Earth’s crust, averaging about 1 mg/kg in rocks and minerals. Because of chemical similarities of Ge and Si, the crustal geochemistry of Ge is dominated by a tendency to replace Si in the lattice sites of minerals.^1^ In natural aquatic systems, dissolved inorganic germanium (1 to 20 ng/kg) behaves like silicon (∼1 to 20 mg/kg) during diatom uptake and dissolution in marine and fresh waters,^2^ thus providing a virtually perfect tracer for biogenic silica cycling in the ocean.^3,4^

Germanium has five naturally occurring isotopes, ^70^Ge, ^72^Ge, ^73^Ge, ^74^Ge, and ^76^Ge, with relative abundances of 21.23, 27.66, 7.72, 35.94, and 7.45%, respectively.^5^ The significant Ge isotope fractionation in the chemical reduction of GeO_2_ to GeO was primary predicted by Brown and Krouse^6^ by calculating partition-function ratios. Despite the interest, due to several limitations in reliable measuring procedures, there are less than a dozen of published studies reporting natural variations of Ge isotopes.^7^ The high ionization energy of germanium prevents the measurement of small samples (<μg) using thermal ionization mass spectrometry (TIMS). Because of this limitation, relatively few investigations of fractionation in terrestrial samples have been made so far. Thus, it was concluded that terrestrial variations of germanium isotopic ratios are restricted to a few per mil.^8−10^ An essential improvement in the development of multicollector inductively coupled plasma mass spectrometry (MC-ICPMS) allowed the progress toward the high-precision measurement of Ge isotopic compositions.^11^ Hirata,^12^ Xue et al.^13^ and Luais et al.^14^ analyzed the Ge isotopic composition of meteoritic materials and identified the first direct evidence for fractionation of Ge isotopes.

Although further analytical developments were made to evaluate inherent instrumental peculiarities regarding Ge isotopes^10^ and to elaborate the isotope analysis method for its application in cosmochemistry,^15^ the natural variations of Ge isotopes on Earth remained unknown, mainly due to the lack of suitable analytical techniques to analyze silicate matrices and submicrogram quantities of Ge.^7^

Much effort to develop new procedures to improve the precision of Ge isotope measurements in geological and aqueous matrices, including silicates and geothermal fluids, was done in recent years.^16−21^ Studies have reported Ge isotope variations in low-temperature Earth surface environments,^16−18^ coal,^22^ and ore deposits,^18,20,23^ as well as crustal rocks and meteorites.^15,16,18^

Before undertaking isotopic analysis by MC-ICPMS, it is mandatory to separate the analyte from both isobaric elements that can potentially interfere with the analyte as well as matrix elements that can affect the mass bias on the instrument and can form complex compounds, also causing an interfering effect with the element of interest.^7^ To achieve this goal, ion-exchange chromatography provides the most versatile and convenient technique. Luais et al.^14,15,24^ adapted the method of Xue et al.^13^ and used cation-exchange resin for the separation of Ge from metallic and sulfide matrices in diluted HNO_3_ medium. A sample solution in 0.5 M HNO_3_ was loaded onto AG50W-X8 cationic resin. The extremely low partition coefficient for Ge (occurring as oxyanion) with 0.5 M HNO_3_ allows the elution of Ge, whereas all the matrix elements (occurring as cations) remain absorbed on the resin. Rouxel et al.^16^ reported a comprehensive chromatography separation procedure that is applicable to a range of geological samples. Despite many efforts to find a suitable way of separating Ge from the matrix, the sample obtained in this way is usually not suitable for direct measurements on MC-ICPMS and it is necessary to use a hydride generation (HG).^7^

The generation of volatile metalloid hydride has long been the most suitable technique for on-line separation and speciation of ng to pg amounts of Ge, As, Se, Sb, and Sn.^25−28^ This procedure involves the reduction of the element of interest in the solution to its volatile hydride species using a strong reducing agent, such as NaBH_4_, generating hydrogen (in status nascendi) upon mixing with acidified sample solution. The separation of the evolved gas and remaining solution is performed using a dedicated hydride generation (HG) system. It is worth mentioning that there is a risk of in situ decomposition of generated hydrides of Ge, Se, etc., in the presence of selected transition metals.^29,30^

Instrumental mass bias is generally corrected using either the standard-sample bracketing (SSB) or the double-spike method. Important advantages of the use of HG-MC-ICPMS are as follows: (1) higher sensitivity, lowering the total amount of element required for one analysis in comparison to sample introduction by nebulization, and (2) further separation of the analyte from its matrix, removing potential isobaric interferences (e.g., Zn).^7^

Germanium isotope measurements by MC-ICPMS suffer from molecular interferences, such as ^35^Cl^35^Cl^+^ on ^70^Ge^+^, ^40^Ar^16^O_2_^+^ and ^36^Ar^36^Ar^+^ on ^72^Ge^+^, ^58^Ni^16^O^+^ and ^38^Ar^36^Ar^+^ on ^74^Ge^+^, and ^38^Ar^38^Ar^+^ and ^36^Ar^40^Ar^+^ on ^76^Ge^+^, in addition to isobaric interferences of ^70^Zn^+^ on ^70^Ge^+^. The chemical purification step and hydride generation technique may remove some of these interferences.^7^ Therefore, it appears that all Ge isotopes, except the less abundant ^76^Ge, can be measured without significant correction for interferences.^10,15,16^

The approach presented in this paper is primarily intended to improve the sample preparation step, as well as the sensitivity and precision of the isotope ratio measurements. Instead of ion-exchange resins, liquid–liquid extraction was successfully used to separate Ge from matrix elements.^31^ Germanium species are being extracted from a highly polar medium of 9 M HCl to less polar organic solvent. Next, they are re-extracted to water. Additional application of acetic buffer solution allows for a significant improvement in sensitivity in comparison to other authors who performed hydride generation in low pH.

The aim of this work is to develop a new procedure for a precise Ge isotope ratio analysis with improved analytical characteristics and to check its applicability on geological standard reference materials (SRMs) for further use in the analysis of real samples.

## Experimental Section

### Reagents and Standards

All chemicals were of analytical reagent grade. All samples and standards were diluted with deionized water (Milli-Q Integral 3 Q-POD Water Purification System, Merck Millipore, Darmstadt, Germany).

Selected geological reference materials were analyzed to validate the proposed analytical procedure. They include U.S. Geological Survey reference materials BHVO-2 (Hawaiian basalt), GH (granite; Hoggar, Algeria), GL-O (glauconite; Normandy, France), and IF-G (iron formation; West Greenland).

Hydrofluoric acid (40%), nitric acid (65%; both Merck Suprapur, Darmstadt, Germany), and phosphoric acid (≥85%; Sigma, Milwaukee, WI, USA) were applied to dissolved geological SRMs.

EMSURE fuming hydrochloric acid (37%) and chloroform for liquid chromatography (both Merck, Darmstadt, Germany) were used in the extraction procedure. Compressed helium (Air Products, Warsaw, Poland) was used to purge samples after extraction.

Sodium borohydride (Sigma, Milwaukee, WI, USA), sodium hydroxide micropills (POCH, Gliwice, Poland), sodium acetate trihydrate (≥98.0%; POCH, Gliwice, Poland), and glacial acetic acid (100%; Merck, Darmstadt, Germany) were used for the generation of germanium hydride. NaBH_4_ solution (1%, w/v) in 0.01 M NaOH was freshly prepared on a daily basis^31^ by dissolving consecutively 0.24 g of sodium hydroxide and 6.0 g of sodium borohydride in 600 mL of deionized water. To prepare the acetic acid–sodium acetate stock buffer solution (1 M), 34 g of CH_3_COONa·3H_2_O was dissolved in ∼150 mL of deionized water, then 4.8 mL of glacial CH_3_COOH was added, and the obtained solution was diluted to 250 mL with water. Acetic buffer solution (0.1 M) was prepared by an appropriate dilution of the stock solution.

Two batch solutions of NIST 3120a standard (LOT 000411 and LOT 151115, both containing 10 g/L Ge) were used in the analysis, but only LOT 000411 was applied as a standard reference material with δ^74/70^Ge equal to 0. Bracket solutions for measuring delta values of Ge via the SSB procedure were prepared by spiking 0.1 M acetic buffer with NIST 3120a solution to a final Ge concentration of 25–80 μg/L, trying to match the intensities of the bracket and the sample.

Single-element nickel and copper ICP standards (both Merck, Darmstadt, Germany), iron atomic absorption standard (VHG Labs, Manchester, NH, USA), and zinc calibration standard (CPAchem, Stara Zagora, Bulgaria), as well as ICP multielement standard solution VI (Merck, Darmstadt, Germany), were used in interference study by spiking the diluted NIST 3120a solution (70 μg/L Ge) to a final content of interfering ions of 0.2–2.0 mg/L. The synthetic seawater was prepared similarly as in ref^32^ by an appropriate dilution of chloride salts of sodium (Sigma, Milwaukee, WI, USA), magnesium, and potassium (both Merck, Darmstadt, Germany) with deionized water with the only difference that sodium sulfate (Sigma, Milwaukee, WI, USA) was also added to the mixture. The solution obtained had a comparable composition to the natural seawater^33^ (Table 1). This synthetic seawater was spiked with NIST 3120a to a total Ge concentration of 80 μg/L.

**Table 1:** Composition of the Synthetic Seawater

component	Cl^–^	Na^+^	Mg^2+^	SO_4_^2–^	Ca^2+^	K^+^
total concentration in natural seawater,^31^ g/L	19.4	10.8	1.27	2.71	0.41	0.40
concentration in synthetic seawater, g/L	18.8	10.8	1.27	2.71	not added	0.40

### Sample Preparation

Dissolution of geological SRMs for Ge isotopic measurements was carried out in closed vessels in agreement with the procedure presented in ref.^19^ A rock (0.5 g) was weighed in a Teflon vessel, then 10 mL of 1:3 (v/v) mixture of HNO_3_ (65%) and HF (40%) was added, and the vessel was closed with a Teflon tap. The digestion was carried out on a hot plate at 60 °C during 48 h. The vessel was cooled down, and its content was transferred into a 15 mL polypropylene centrifuge tube (VWR, Radnor, PA, USA) and centrifuged, with the supernatant transferred into a Teflon beaker. The solid residue was then leached with 0.5 mL of HF (40%), and after being vortexed for about 1 min, the mixture was centrifuged again, with the supernatant added to the previously collected supernatant. Leaching and centrifugation steps were repeated three times. Then, the combined supernatant solution was evaporated at 60 °C until a gel was obtained. The gel was then redissolved in 2 mL of concentrated nitric acid and then dried down. Finally, the sample residue was dissolved in 25 mL of 1% HNO_3_ for storage. Solutions obtained according to this dissolution procedure were analyzed by Q-ICPMS (NexION 300D, PerkinElmer, Waltham, MA, USA) for Ge contents.

Two alternative procedures of geological standard digestion were also checked to find the most suitable one. The first one was similar to the procedure described above, but the dissolution was carried out in opened vessels with a magnetic stirrer at room temperature. The second alternative method with the application of phosphoric acid was adopted from our previous work.^31^

Moreover, a few thermal water samples (KT-1, C-1, DM-2, DM-5, and DM-7) from the Sudetes mountain range (Southern Poland), which are relatively rich in Ge, were analyzed for their isotope ratio of Ge. The content of Ge in those water samples was approximately 5 μg/L, so the preconcentration step was necessary. About 500 mL was evaporated in Teflon vessels on a hot plate at 55 °C. The evaporation was continued until about 8 mL of the sample was left. Next, the concentration of Ge was controlled using Q-ICPMS. To validate this procedure, also a sample containing about 150 mL of deionized water spiked with NIST SRM 3120a to a Ge concentration of 10 μg/L was proceeded the same way.

The next step was the extraction of Ge from solutions and preparations for measurements, which were carried out according to the previously reported procedure^31^ (with some modifications). Briefly, the procedure was as follows:

(1) Twenty-five milliliters of the sample solution was mixed with 75 mL of fuming hydrochloric acid in a 100 mL separatory funnel.
(2) Fifteen milliliters of chloroform was added to the funnel.
(3) The mixture was shaken for 2 min.
(4) The chloroform extract was transferred into a 50 mL test tube.
(5) Steps 2–4 were repeated twice.
(6) The aqueous phase with acid was carefully removed from the funnel, which in turn was properly rinsed with deionized water.
(7) The combined chloroform extract (45 mL) was transferred back to the separatory funnel, with 5 mL of water added to it.
(8) Actions 3 and 4 were carried out.
(9) The aqueous re-extract was poured into a clean test tube.
(10) Steps 7–9 were repeated twice;
(11) A PTFE tube from the helium bottle was placed in a test tube with 15 mL of the obtained water re-extract; the solution was purged with helium for about 1 h in the way that the bubbles coming out from the sample could be counted.
(12) A 1 M acetic buffer solution (2.5 mL) was added to the sample, and the obtained solution was diluted with water to a total volume of 25 mL. The sample was ready for analysis, but it is strongly recommended to purge it with He for at least 5 min before every consecutive measurement.

### HG-MC-ICPMS Analysis

Germanium isotope ratios were measured at the Biological and Chemical Research Centre of the University of Warsaw using the “Plasma II” MC-ICPMS equipped with 16 Faraday cups (Nu Instruments, Wrexham, UK). The amplifier boards of the collectors were calibrated on a daily basis using an internal 40 V reference signal. Fine-tuning of the MC-ICPMS instrument was performed before each measurement session. The HGX-200 advanced membrane hydride generation system (CETAC Technologies, Omaha, NE, USA) was applied for sample introduction. Time-resolved analysis (TRA) mode was used for gathering experimental data as it proved to be a better measurement approach when transient signals are considered.^32,34^ TRA also enables for individual real-blank correction of each registered signal, which allows for elimination of Ar-derived molecular interferences. Operating parameters for the HG-MC-ICPMS system are listed in Table 2.

**Table 2:** HG-MC-ICPMS Operating Parameters

MC-ICPMS parameters	
RF power	1300 W
coolant flow (Ar)	13 L/min
auxiliary flow (Ar)	1.15 L/min
nebulizer gas flow (Ar)	0.69 L/min
interface cones	nickel
	
measurement parameters	
resolution mode	∼300
cup configuration	H4: ^74^Ge; H2: ^73^Ge; Ax: ^72^Ge; L4: ^70^Ge
integration time	2.5 s
measurement mode	time-resolved analysis
replicates	≥50
	
hydride generation	
“acid” reagent	0.1 M sodium acetate buffer
reducing agent	NaBH_4_ (1%) mixed with NaOH (0.01 M)
reagents and sample flow rate	0.83 mL/min
sample gas rotameter position	10 mm
additional gas flow rate	0.7 L/min

The determination of Ge isotopic changes was carried out using the SSB method by sequential measurements of the standard-sample-standard (procedure 1). Such external calibration with a standard (NIST SRM 3120a) provides the delta value, calculated according to eq 1:
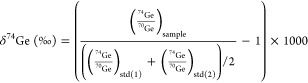
1

To confirm the applicability of the hydride generation system for measurements of Ge isotope ratios on MC-ICPMS, a desolvation nebulizer (Aridus II, CETAC Technologies, Omaha, NE, USA) was also applied and the results from two sample introduction units were compared. For this purpose, the solution of NIST SRM 3120a was used without sample pretreatment or any additional matrix. The calculation of Ge delta values was also carried out using the SSB method (procedure 2). Moreover, a desolvation nebulizer enabled the application of the internal standard (IS) technique, in which Ge solution (400 μg/L) was spiked with 280 μg/L Ga (WZORMAT, Warsaw, Poland), which served as an internal standard. ^69^Ga and ^71^Ga isotopes were measured on detectors L5 and L2, respectively, and the calculations were done in the same way as in ref^32^ (procedure 3).

## Results and Discussion

### Validation of the Experimental Procedure

The first task of this research was to connect the HG sample introduction system with the MC-ICPMS instrument and obtain as high signal intensity for Ge as possible by optimizing the parameters as in Table 2. The sensitivity of the HG-MC-ICPMS method for Ge isotope ratio determination is directly proportional to the sample flow rate that was applied. However, too high sample solution throughput results in a less stable analytical signal and sample overconsumption, so the compromise value was chosen. Ultimately, the sensitivity of the proposed HG-MC-ICPMS method under optimal experimental conditions (40 V for ^74^Ge in 100 μg/L solution) is higher than it was later obtained with the application of a desolvation nebulizer (10 V/100 μg/L).

The solution of the NIST SRM 3120a standard was measured on MC-ICPMS after sample introduction using a HG system without any pretreatment (neither evaporation nor extraction). The δGe values were obtained using the SSB method. Measurement conditions as in Table 2 were optimized until the mean δGe for three isotope pairs (^74^Ge/^70^Ge, ^74^Ge/^72^Ge, and ^74^Ge/^73^Ge) reached values close to zero with the two standard deviation (2SD) values around 0.1‰, which in turn corresponds to the precision of MC-ICPMS instrument.

Two batches of NIST SRM 3120a (LOT 000411 and 151115) were available for measurements. Interestingly, LOT 151115 measured relative to LOT 000411 gave the delta value that differed from zero significantly (Table 4). This batch was analyzed with three different procedures, and each approach gave a delta value other than zero. Description of these procedures is in Table 3. Nevertheless, we report that there is a difference in isotopic composition of Ge between two batch solutions of the same NIST 3120a standard. That is why one should be careful with reporting the results of Ge isotope ratio analysis measured regarding NIST SRM 3120a. It seems that any reported δGe isotope ratio value could not be taken into consideration without mentioning the exact batch of the standard (LOT 000411 or 151115) that was used as a reference material.

**Table 3:** Procedures Used for Measurements of Isotopic Composition of Two Batches of NIST SRM 3120a

procedure number	sample introduction	calibration
1	hydride generation	SSB
2	desolvation nebulizer	SSB
3	desolvation nebulizer	IS-SSB

Procedure 1 is the hydride generation SSB method. Procedure 2 is SSB with a desolvation nebulizer as a sample introduction (NIST SRM does not contain any matrix, so separation is not necessary). Procedure 3 is the internal standard combined with SSB (IS-SSB), where isotopes of gallium were used as an internal standard. In this procedure, the sample was introduced using a desolvation nebulizer (exactly as it was in procedure 2). In our daily routine, we usually try to match the concentrations of samples and bracket as accurately as possible. However, our experience shows that a 25% difference does not reduce the quality of the results.

According to the results provided in Table 4, two SSB methods (procedures 1 and 2) with different ways of sample introduction (desolvation nebulizer and hydride generator) led to correct δGe values for LOT 000411 alone and close δGe values for LOT 151115, but in most cases, the precision was a bit worse when the HG unit was applied (higher 2SD values). On the other hand, the IS-SSB method (procedure 3) provided results that slightly differed from the previous two for the second batch solution, despite a good precision.

**Table 4:** δGe Isotope Ratio Values of Two NIST SRM 3120a Batch Solutions Measured with Different Methods Regarding LOT 000411 (*n* – Number of Replicates)

		isotope ratio						
		^74^Ge/^70^Ge		^74^Ge/^72^Ge		^74^Ge/^73^Ge		
NIST 3120a batch	procedure	δ, ‰	2SD	δ, ‰	2SD	δ, ‰	2SD	*n*
000411	2^a^	0.00	0.04	0.00	0.02	0.00	0.02	4
151115	2^a^	–0.33	0.05	–0.16	0.03	–0.07	0.03	6
000411	3^a^	0.00	0.07	0.00	0.04	0.00	0.05	4
151115	3^a^	–0.25	0.09	–0.12	0.05	–0.05	0.04	6
000411	1^b^	–0.01	0.09	0.00	0.09	–0.02	0.15	9
151115	1^b^	–0.34	0.06	–0.17	0.05	–0.08	0.04	8

a Desolvation nebulizer.

b Hydride generation system.

In this study, only batch 000411 was used as a reference material because, in most cases, previously reported research studies also applied this standard solution. A desolvation nebulizer (procedures 2 and 3) was not used in further investigations as it does not provide satisfactory matrix removal.

**Figure 1:**
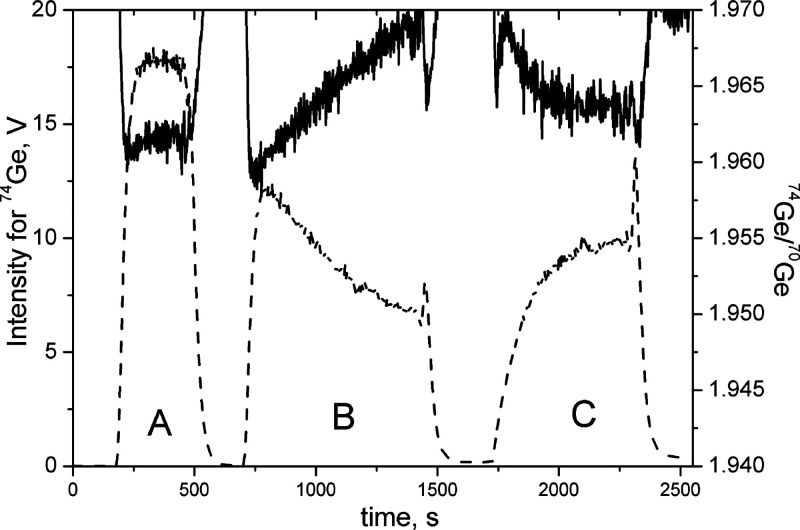
Intensity for ^74^Ge (dashed line) and isotopic ratio ^74^Ge/^70^Ge (solid line) used in the isotopic analysis of NIST SRM 3120a: the Ge intensity signal in the middle (B) corresponds to NIST SRM 3120a solution with the addition of transition metal ions; signals to the left and right (A, C) refer to pure Ge NIST SRM 3120a solution (brackets).

As it was mentioned above, transition metals are the most common reason of an interfering/decomposition effect while using the HG system. The addition of selected transition metals (Cr, Fe, Ni, Cu, and Zn, 200 μg/L each) to the NIST SRM 3120a solution caused the gradual decrease in the Ge signal and led to large changes in the isotopic ratio of Ge, as it can be seen in the case of the middle signal in Figure 1. Moreover, the second bracket of a pure NIST SRM 3120a solution (signal to the right) was still affected by the presence of impurities. The hydride generation installation requires a complete and thorough cleaning sequence after measuring such samples. This adverse effect is usually limited by the addition of chelating agents such as cysteine, but on the other hand, this makes the matrix more complicated. For this reason, extraction of Ge with chloroform seems to be a great solvation of this problem due to its specificity toward Ge.

In a previous work,^31^ it was shown that the extraction efficiency of Ge with carbon tetrachloride after its reaction with concentrated hydrochloric acid is higher than 99%, as the corresponding value for other metals is below 1%. In the present study, chloroform was used instead of CCl_4_ due to environmental safety precautions and also investigated in terms of Ge extraction efficiency. For that purpose, the ICP multielement standard solution was added to the NIST 3120a standard solution and Ge was extracted according to the proposed procedure.

Results in Table 5 evidently show that most of other metals that were investigated besides Ge are extracted to chloroform with the yield less than 1%. In such a way, the possible influence of transition metals, such as Cr, Fe, Ni, Cu, and Zn, was limited. Moreover, the influence of isobaric interferences (^70^Zn and ^74^Se) is also eliminated.

**Table 5:** Extraction Efficiency of Ge and Different Possible Interfering Metal Species with Chloroform

metal	added, μg/L	found, μg/L	extraction yield, %
Ge	200	>190	>95
Li	200	0.10	0.05
B	2000	1.92	0.10
Al	200	2.65	1.33
Cr	200	1.03	0.52
Mn	200	<0.1	<0.05
Fe	2000	6.97	0.35
Co	200	<0.1	<0.05
Ni	200	0.84	0.42
Cu	200	1.27	0.64
Zn	2000	6.27	0.31
As	2000	6.00	0.30
Se	2000	19.6	0.98
Mo	200	<0.1	<0.05
Cd	200	<0.1	<0.05
Ba	200	<0.1	<0.05
Tl	200	<0.1	<0.05
Pb	200	0.14	0.07

### Ge Isotope Measurements of SRMs and Natural Water Samples

**Figure 2:**
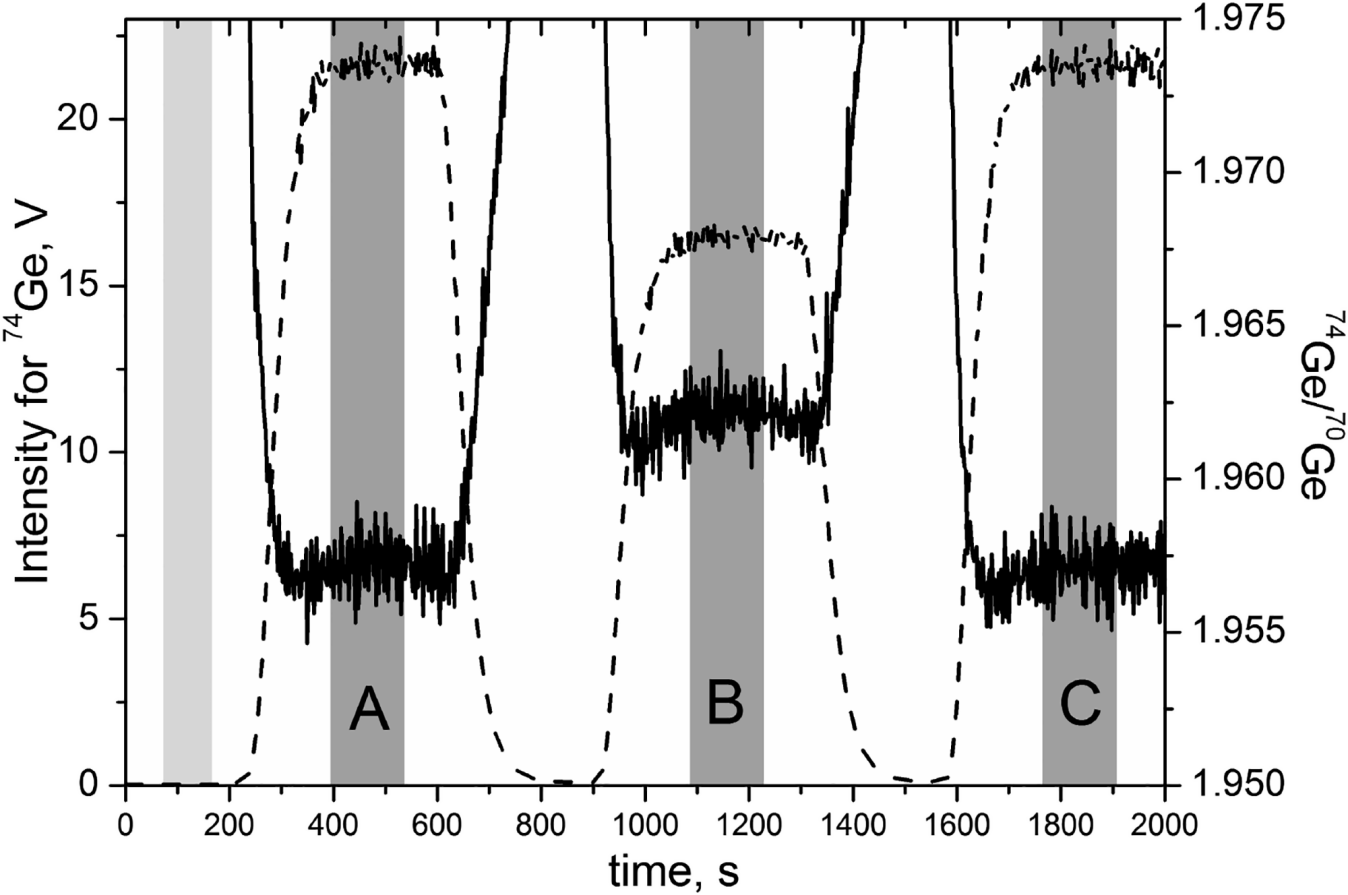
Intensity for ^74^Ge (dashed line) and isotopic ratio ^74^Ge/^70^Ge (solid line) in the isotopic analysis in GL-O SRM: the signal in the middle (B) corresponds to the sample solution; signals to the left and right (A, C) refer to pure Ge NIST SRM 3120a solution (brackets); light gray and gray areas represent the time periods when signals from blank and Ge solutions, respectively, were integrated.

To check the applicability of the proposed HG-MC-ICPMS method for Ge isotope analysis after extraction with chloroform, Ge isotope ratios were measured in standard reference material NIST 3120a solutions, synthetic seawater samples spiked with NIST SRM 3120a, and geological reference materials (Table 6). The example of an analytical signal registered in the time-resolved mode is shown in Figure 2. The flat-top peaks were integrated for at least 2 min to calculate Ge isotope ratios and then obtain delta values after further processing according to eq 1.

**Table 6:** δ^74/70^Ge Isotope Ratio Values Measured Regarding NIST SRM 3120a (LOT 000411) Using HG-MC-ICPMS with SSB Correction

sample	δ^74/70^Ge, ‰	2SD	*n*	expected δ value
NIST SRM 3120a (LOT 000411), extracted	0.02	0.07	6	0
NIST SRM 3120a (LOT 000411), evaporated and extracted	0.01	0.16	3	0
synthetic seawater spiked with NIST SRM 3120a (LOT 000411)	–0.03	0.12	2	0
USGS BHVO-2 basalt (reported dissolution method)	0.51	0.05	3	0.51^18^
USGS BHVO-2 basalt (alternative dissolution method 1)	0.53	0.02	3	0.51
USGS BHVO-2 basalt (alternative dissolution method 2)	0.67	0.05	4	0.51
IF-G iron formation	1.03	0.06	4	1.03
GH granite	0.73	0.02	3	0.68^18^
GL-O glauconite	2.47	0.05	3	2.44^18^
KT-1 thermal water	0.93	0.17	4	
C-1 thermal water	1.66	0.05	3	
DM2 thermal water	1.17	0.19	3	
DM5 thermal water	1.16	0.10	4	
DM7 thermal water	1.03	0.02	2	

First of all, the results in Table 5 confirm that neither extraction nor evaporation of the sample causes Ge fractionation, as δ^74/70^Ge values for NIST SRM 3120a solutions remain close to zero after such sample preparation. Synthetic sea water spiked with NIST 3210a gave δ^74/70^Ge close to zero, which proves that the Ge isotopic composition of real seawater samples can be successfully measured by the proposed method.

As for the geological standard reference materials, it is complicated to transfer Ge from the solid to a solution. Germanium is known to evaporate during the dissolution of minerals with nitric and hydrofluoric acids even at moderate temperatures (50–60 °C), so the digestion needs to be carried out in closed vessels.^7,18,19^ To avoid the undesirable phenomenon of Ge evaporation that can lead to isotopic fractionation in the sample, different dissolution techniques were checked: a previously reported one,^19^ as well as two alternative methods, both with opened vessels.

The analysis of geological standard USGS BHVO-2 after the digestion according to the procedure reported in the literature led to the expected δ^74/70^Ge value. The first alternative method also provided a good result, so minerals actually can be digested in open vessels. But this can be done only at room temperature, because for the second alternative method, the increase in the temperature caused some Ge isotope fractionation. The results for other mineral standards (IF-G, GH, and GL-O) were also in close agreement with the literature values when the optimal dissolution procedure was used (Table 5).

After the proposed HG-MC-ICPMS procedure was checked on various standard reference materials, it was applied for the Ge isotope analysis of selected water samples. As can be seen in Table 6, the samples marked with DM and KT had a relatively close isotopic composition. On the other hand, the difference between the δ^74/70^Ge values of sample C-1 was much more significant.

## Conclusions

In this study, a new sensitive procedure for the accurate and precise measurements of the Ge isotope ratio in synthetic water and natural samples of geological origin by MC-ICPMS using an on-line HG system was developed. The main advantage and novelty of this method were the application of liquid–liquid extraction of Ge to eliminate all elements depressing the generation of germanium hydrides with no need of laborious Ge preseparation with ion-exchange resins. We proved that neither extraction nor evaporation of samples caused isotopic fractionation, which was an essential experimental evidence enabling further investigations. Other advantages of the proposed method are as follows: (1) the best possible matrix removal achieved using a HG system; (2) a relatively high sensitivity, lowering the total amount of Ge in the sample required for a precise isotope analysis down to 120 ng, which can be additionally enhanced by using a simple preconcentration procedure.

The applicability of the proposed method was confirmed by obtaining delta values of the ^74^Ge/^70^Ge isotope ratio of standard reference materials close to those reported in the literature. The application of the standard-sample bracketing method proved to be a useful way to correct the instrumental bias. The substitution of a HG system for a desolvation nebulizer and the combination of SSB with the internal standard method (IS-SSB) did not lead to a significant improvement of results. Furthermore, different techniques of geological sample dissolution were checked. It is recommended to digest samples in closed vessels to prevent any Ge loss and isotopic fractionation related with it.

Furthermore, we report that there is a difference in isotopic composition of Ge between two batch solutions of the same NIST 3120a standard.

As the new method of Ge isotope ratio determination is simple and quick, it has been already successfully applied for the isotope analysis of natural water samples with a low content of germanium.
